# How Change of Public Transportation Usage Reveals Fear of the SARS Virus in a City

**DOI:** 10.1371/journal.pone.0089405

**Published:** 2014-03-19

**Authors:** Kuo-Ying Wang

**Affiliations:** Department of Atmospheric Sciences, National Central University, Chung-Li, Taiwan; Kantonal Hospital St. Gallen, Switzerland

## Abstract

The outbreaks of the severe acute respiratory syndrome (SARS) epidemic in 2003 resulted in unprecedented impacts on people's daily life. One of the most significant impacts to people is the fear of contacting the SARS virus while engaging daily routine activity. Here we use data from daily underground ridership in Taipei City and daily reported new SARS cases in Taiwan to model the dynamics of the public fear of the SARS virus during the wax and wane of the SARS period. We found that for each reported new SARS case there is an immediate loss of about 1200 underground ridership (the fresh fear). These daily loss rates dissipate to the following days with an *e*-folding time of about 28 days, reflecting the public perception on the risk of contacting SARS virus when traveling with the underground system (the residual fear). About 50% of daily ridership was lost during the peak of the 2003 SARS period, compared with the loss of 80% daily ridership during the closure of the underground system after Typhoon Nari, the loss of 50–70% ridership due to the closure of the governmental offices and schools during typhoon periods, and the loss of 60% daily ridership during Chinese New Year holidays.

## Introduction

The 2003 SARS epidemic is a recent vivid example, demonstrating the deep impact that a deadly virus can have on a society. For example, TIME magazine called Taiwan a SARS Island [1], that SARS sinks Taiwan [2], and China as a SARS Nation [3]. The causative agent for the SARS disease was found to be a novel coronavirus, originated in bats and infected people most likely through wild animal markets [4]–[6]. The first known SARS case was a 45-year-old man in Foshan, Guangdong, China, in November 2002. Hong Kong was the first place for the global diffusion of the SARS virus when a 64-year-old nephrologist from Guangzhou, Guangdong, checked into room 911 of the Metropole Hotel in Kowloon in the night of 21 February 2003, checked out the next morning, been admitted to the Hong Kong Prince of Wales Hospital where he died several days later. Sixteen hotel guests and one visitor who stayed at the hotel on that night contracted the SARS virus, and carried the virus to Hanoi, Toronto, and Singapore.

**Figure 1:**
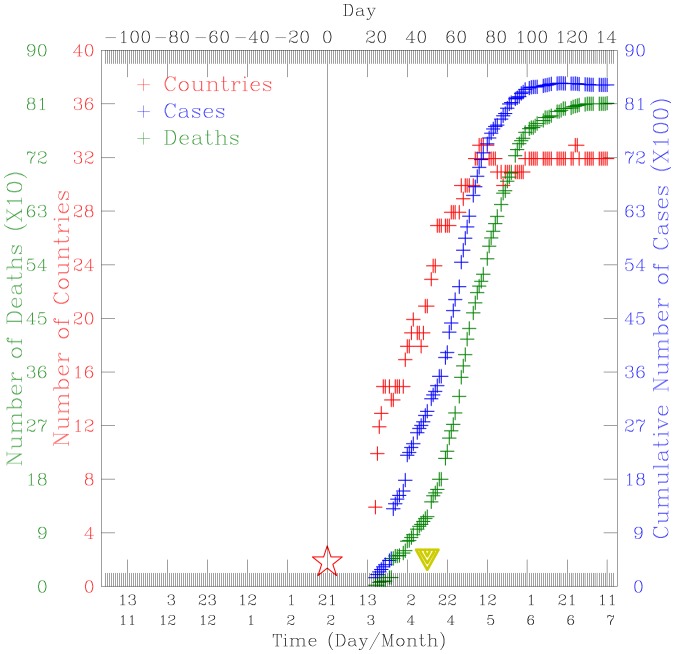
Time-series plot of the accumulated number of countries with reported SARS cases (red crosses), daily global reported cases (blue crosses), and deaths (green crosses) during the period 1 November 2002–15 July 2013. The red open star indicates the arrival of the 64-year-old nephrologist in Hong Kong on 21 February 2003. The inverted green triangle indicates the day when the first SARS coronavirus was sequenced. Data are compiled from the World Health Organization (www.who.int/csr/sars/country/en/, accessed 3 December 2013).

Figure 1 shows a time series plot of the spatial diffusion of the epidemic as the cumulative number of countries affected, daily reported SARS cases, and deaths. It takes the SARS virus about 80 days to spread over more than 30 countries, 100 days to infect more than 8000 people, and 120 days to cause about 800 deaths, a fatality ratio of about 1 in 10 [6]. The hardest hit regions are China (5327 cases, 349 deaths), Hong Kong (1755 cases, 299 deaths), Canada (251 cases, 43 deaths), Taiwan (346 cases, 37 deaths), and Singapore (238 cases, 33 deaths) (WHO, 2003). The fatality ratios for these hardest regions ranging between 7% (China), 11% (Taiwan), Singapore (14%), and 17% (Canada, Hong Kong) [7]. The first recognized SARS case in Taiwan was a 54-year-old businessman who traveled to Guangdong, China, on 5 February 2003, and returned to Taiwan via Hong Kong on 21 February. He had developed fever, myalgia, a dry cough but was not hospitalized until 8 March 2003 [8]. It is unknown if this Taiwanese businessman had flew the same airlines with the Chinese nephrologist from Guangzhou because the day of their arrival in Hong Kong is the same, 21 February 2003. The wife and the son of this Taiwanese businessman were later hospitalized (on 14 and 21 Mar, respectively) and developed the novel SARS coronavirus. Most of the early cases in Taiwan were imported from China and Hong Kong [9]. We note that on 10 July 2003, there were 671 probable cases and 84 deaths. However, since 11 July 2003, 325 cases have been discarded. Laboratory information was insufficient or incomplete for 135 discarded cases, of which 101 died [7]. According to the World Health Organization (WHO) statistics [7], 4.2% of global cases and 4.8% of global deaths had occurred in Taiwan. About 63% of the SARS cases were female, and 37% cases were male, and the median age for these cases is 42 (ranges 0–93) in Taiwan [7]. The reported SARS cases were concentrated in Taipei City and Taipei County (now called New Taipei City) [9]. About 71% of probable cases were located in Taipei City and Taipei County [10]. Both Taipei City and Taipei County are linked by the same underground massive transportation system. Almost 73% of all traceable infections in Taiwan occurred in hospital settings [11]. Started on 14 March 2003, when the first SARS case was recognized, Taiwan moves aggressively to isolate all suspected or probable SARS cases in negative-pressure rooms in hospitals [8]. Contacts of known patients, both suspected and probable cases, were strictly put into quarantined at home for 10 days since 28 Mar 2003. These include those healthcare workers exposed to outside isolation settings, family and other close contacts, those on the airplanes with SARS patients (seated two in front of or three rows behind a patient) [8]. The majorities of cases occurred after 21 April 2003, and were associated with transmission in health-care settings [10]. All probable SARS patients were hospitalized [10], [12].

Previous works studied the dynamics of the daily accumulated infected cases during the SARS outbreaks in Beijing, Canada, Hong Kong, Singapore, and Taiwan [11], [13], [14] respectively. However, there is a lack of study to quantify the change of people's behavior resulting from the fear incurred by daily reported SARS cases. Moussaïd [15] described how the feeling of fear and the perception of danger can propagate from one individual to another in large populations of interacting people, giving rise to large-scale behavioral patterns such as avoidance of public transportation. As such, one method to measure people's fear of the SARS virus is by studying the change of people's daily activity with respect to the variations of the daily reported SARS cases during the epidemics. Because a confined environment with people stay in short distance with each other is conducive for infectious transmission between people [16], [17], people were advised to stay away from any confined space during the period of the SARS epidemics. This perception of high risk associated with contacting the SARS virus alters people's daily activity. In this work, we use the Taipei underground (subway) mass transportation system, which is a typical of confined space, and reported SARS cases in Taiwan, to show that people's fear of the SARS virus can be quantified by a combination of the fresh fear (incurred by the daily announced SARS cases) and the residual fear (dissipation of the fresh fear produced in previous days).

## Data and Methods

### 1. Daily Underground Ridership Data

The Taipei underground system transports about 1 million people per day during the 2001–2005 period [27].

These daily ridership exhibits a strong weekly cycle. A lower amount of people traveling on Wednesday (a short weekend), a weekly peak on Friday (before the long weekend), the lowest amount of people traveling on Saturday and Sunday, and the rest of the week days are about the same. Except for occasional events such as typhoon and the Chinese New Year [18], the weekly pattern is roughly the same through the year. This stability in the daily ridership provides a good quantifiable measure of public fear when an unprecedented and deadly virus occurs in the society.

### 2. Daily Reported SARS Cases Data

After the first SARS case was confirmed on 14 Mar 2003, Taiwan moved aggressively to isolate all suspected or probable case-patients in negative-pressure rooms in hospitals [8]. A total of 3032 suspected or probable SARS cases were reported before 5 July 2003. Among these cases, 664 cases were classified as probable SARS cases based on the clinical case definition (using polymerase chain reaction [10]), and 346 cases had a positive results for the SARS coronavirus [28]. The daily probable cases, according to clinical case definition, are used in this work. Also, the government had attempted to place more than 150,000 people under home quarantine. Level A quarantine was started on 18 Mar 2003, aimed at people having close contact with a suspected SARS case-patient. Level B quarantine was started on 28 April 2003, after the first SARS death on 26 April in Taiwan, aimed at those who traveled from affected areas [26].

The real-time reported probable SARS cases [29], [12] were used in this work. These were the information affecting people's decision during the height of the SARS epidemics. The SARS cases published after the SARS epidemics are slightly different [10], [11], [30]. The data used in this work is similar to the data shown in US CDC report [12]. This data set contains a longer and more complete period of data (from late February to early July, 2003) than those of Hsieh et al. [11] (data period 22 April - 4 June 2003), US CDC [10] (data period late February - late May 2003), and Chen et al. [30] (data period 15 March - 12 May 2003).

### 3. A Statistical Model for Ordinary Daily Ridership

Since weekly patterns of the passengers are less perturbed during the weeks from early spring to early summer than other periods, we can determine mean daily underground ridership 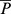 in a weekday based on the average of the 12 weeks, starting from the week with the first Monday in March, for the years 2001, 2002, 2004, and 2005, respectively. Because year 2003 was the year of the SARS epidemic, the underground ridership in 2003 is compared to the dynamics of underground ridership two years before (2001 and 2002) and two years after (2004 and 2005).

Let 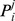 denotes daily ridership in a weekday *i* in year *j*, started from first Monday in March of each year; and *j* is for each of year 2001, 2002, 2004, and 2005.

The average daily ridership 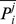 for the same weekday *i* summed over the 12 weeks of year *j* is computed as
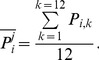
(1)


Here *k* is the week number, started from first Monday in March, and summed for 12 weeks. Equation 1 is systematically used to compute average daily ridership for each weekday of Monday, Tuesday, etc, respectively.

The statistical daily ridership 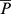 for each weekday in 2003 is modeled as the mean of the daily ridership from 2001, 2002, 2004, and 2005. Therefore,
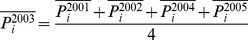
(2)


### 4. A Model for Fresh Fear and Residual Fear

In order to model the daily variations of fear of the underground ridership with respect to the daily reported SARS cases, a dynamical model was developed to simulate day-to-day variations of the underground ridership in the periods before, during, and after the SARS epidemics.

Since people's fear is dynamic in nature, the model variables and the external forcing that governs the time evolution of the model variables must be established so that the model is able to make prediction based on the change of external forcing. Here the external forcing is clearly incurred by the daily reported SARS cases.

During the 2003 SARS period, we observed two significant relationships between the daily underground ridership and the daily reported SARS cases. Firstly, there exists a quick response of the underground ridership with respect to the daily reported SARS cases that made headlines almost everyday in the mass media during the SARS period. The overwhelming reports from these public media appear to have big impacts on the willingness of the public in using the underground as a mean for going to schools and offices (both schools and offices were not closed during the SARS period). The public fear of contacting the SARS virus during the use of the underground system vividly reflected in the significant drops of underground ridership. Secondly, the gradual increases in underground ridership during the final stage of the SARS epidemic. This indicates the return of public confidence in using the underground system as a mean for daily transportation to offices and schools. These two observations indicate that a dynamic model should represent these effects, i.e., the sharp drops in the ridership associated with increases in the reported SARS cases, and a gradual return of the underground ridership as the reported SARS cases gradually faded away from the headlines.

Let 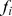 denotes loss of underground ridership due to fear of contacting the SARS virus at day *i*. From the observation of daily ridership behavior, we hypothesize that the fear generated at day *i* will be gradually faded away in the following days. Hence, we can write
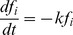
(3)


Here *k* is a constant, representing decay frequency (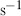) of 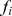. The analytical solution for Eqn. 3 is
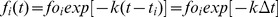
(4)


Here 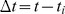. At time 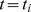,
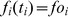
(5)


Here 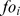 means each new fresh fear and hence new loss of underground ridership generated at day *i* due to a new announcement made at day *i* on the total number of the SARS cases at this day. We call 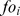 as the fresh fear at day *i*.

Since 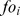 gradually decays away from day *i* following Eqn. 4 for time 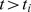, we call 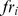 as the residual fear.

Therefore, the residual fear 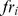 at day *i* is the accumulations of the residual fears dissipated from previous days 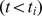,
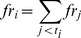
(6)


Hence, at any day *i*, the total fear 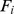 is a summation of a fresh fear 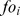 plus the residual fears 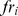 from previous days,
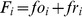
(7)


### 5. A Model for Daily Underground Ridership

Equation 7 quantifies a loss of daily ridership (in the units of people) in day *i* due to fear of contacting the SARS virus. Based on Eqn. 7 and Eqn. 2 the daily underground ridership 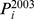 for each day *i* in 2003 is written as
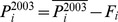
(8)


Hence, the amount of passengers at each day 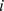 is determined by the daily normal ridership 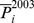, subtracted the loss of ridership due to the number of reported SARS cases at day *i* (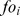) and the accumulated impacts from the days before (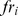).

Since 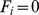 for each day in 2001, 2002, 2004, and 2005. Hence, the daily underground ridership in these non-SARS year is written as
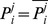
(9)


Here the 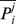 obtained from Eqn. 1 is used to model daily ridership in 2001, 2002, 2004, and 2005. Hence, the daily ridership will normally maintain a constant pattern throughout the week if no other significant factors such as an approaching typhoon, a long holiday, festivals, and epidemics exist.

### 6. Parameters 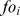 for Fresh Fear and *k* for Residual Fear 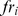


From Eqn. 5 we define fresh fear 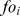 at day *i*. We parameterize 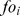 as
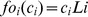
(10)


Here 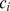 is a variable (the daily reported SARS cases), and *L* is a fixed parameter, representing the loss of underground ridership for each reported SARS case. *L* is empirically determined by comparing model results with the actual underground daily ridership in 2003.

From Eqn. 4, it can be determined that when
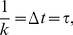
(11)


then
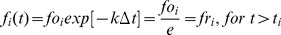
(12)


This indicates that instantaneous passenger loss (fresh fear 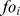) on day 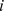 dissipates exponentially to the following days with an 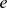-folding time of 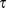 days. Here the *e*-folding time measures the dissipation of the fresh factor (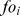) due to each new reported SARS cases (residual fear, 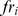), reflecting the public expectation of the risk of contacting the SARS virus arises from each newly reported SARS cases. In this work we also empirically determined *k* by comparing model results with daily underground ridership in 2003. In a similar way to atmospheric chemistry [25], we can call 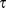 as the lifetime for the fresh fear or the resident time of the fresh fear in the mind of the people.

We note that previous work has shown that effects of respectively epidemic mortality and morbidity on social phenomena can be different [31]. In this work we haven't included the number of deaths in the model. The first death occurred on 26 April (Julian Day 116) [26], which is within the period (22 April, Julian Day 112 - 1 May, Julian Day 121) when the probable cases more than tripled, from 28 to 89 [10]. The source of these outbreaks was due to transmissions occurred in the Taipei City Hoping Hospital [11]. To what extent mortality has an (in)dependent effect on riderships or to what extent morbidity has an independent effect above and beyond mortality remains to be explored.

We also note that, as the model for this work does not consider the effect of the lethality, the model assumes that any news about the increasing or decreasing lethality and cases/deaths from other countries does not affect fear and ridership in Taipei. It remains to be explored in the model the effect of the weekly global number of new cases, deaths, and information on the possible change of lethality over time in addition to the data on Taiwan and Taipei.

## Results

### 1. The Underground Ridership During the non-SARS Years

**Figure 2:**
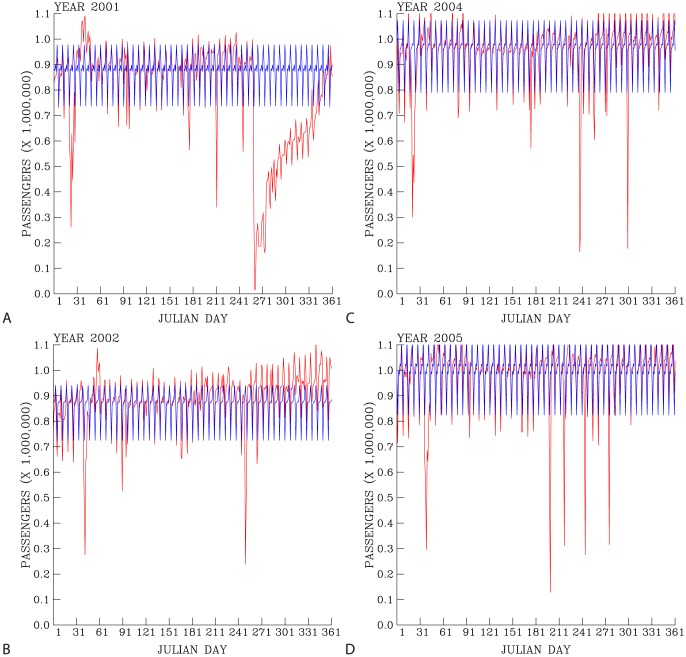
Daily underground passengers (red curve), and the passengers predicted by the statistical model (blue line) in 2001 (A), 2002 (B), 2004 (C), and 2005 (D).

Figure 2 shows a time-series plot of recorded and modeled daily underground ridership during the non-SARS years of 2001, 2002, 2004, and 2005, respectively. During these years, the daily underground ridership can be modeled by the statistical average daily ridership (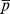) of each year, indicating that the underground usage during normal days are very regular. However, there are days when the model and the actual underground ridership show big discrepancies. These are the days when special events (e.g., Chinese New Year, spring holiday, and typhoons) occurred. For example, Figure 2B shows a typical example of the time-series plot of daily ridership using the Taipei underground system in 2002. We note that the first big drop in ridership after day 31 corresponds to the Chinese New Year holiday, the second big drop in numbers after day 91 is due to students' spring holiday, and the drop after day 241 Julian is due to a typhoon. Similar situations applied to other years as well. We note that a big drop of ridership during the days 241–271 in 2001 is due to the closure of two main lines of the underground system, which was flooded by the severe rainfall during the passing of Typhoon Nari [18].

**Figure 3:**
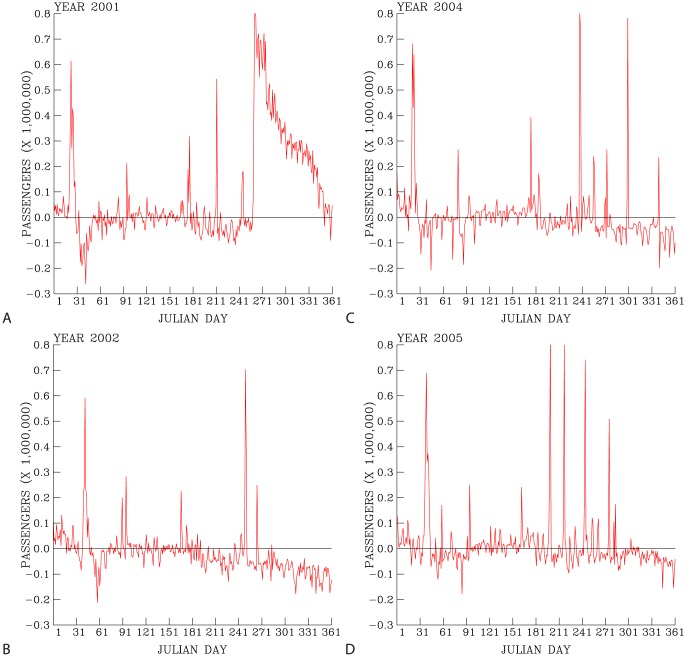
Difference in daily ridership between the model prediction and the actual daily ridership in 2001 (A), 2002 (B), 2004 (C), and 2005 (D).

Figure 3 further compares the discrepancies in ridership between the statistical predictions and the actual numbers. During the spring months (days 61–150) of each year, the statistical model predicts the daily ridership that are, in most of the days, close to within 10% of the actual numbers of people taking the underground. Exceptions occur during rare events (e.g., long holidays, festivals, typhoons, etc). We find that the underground flooding in 2001, caused by Typhoon Nari, resulted in a loss of about 80% daily ridership; the Chinese New Year holiday causes a loss of 60–70% daily ridership; and the close of the governmental offices and schools during typhoon periods caused a loss of 50–80% daily ridership. The increases in people taking the underground toward the end of 2002, 2004, and 2005, respectively, is due to the factors that the underground ridership was steadily growing and also more people tend to take underground during the winter months.

### 2. The Daily Reported New SARS Cases

**Figure 4:**
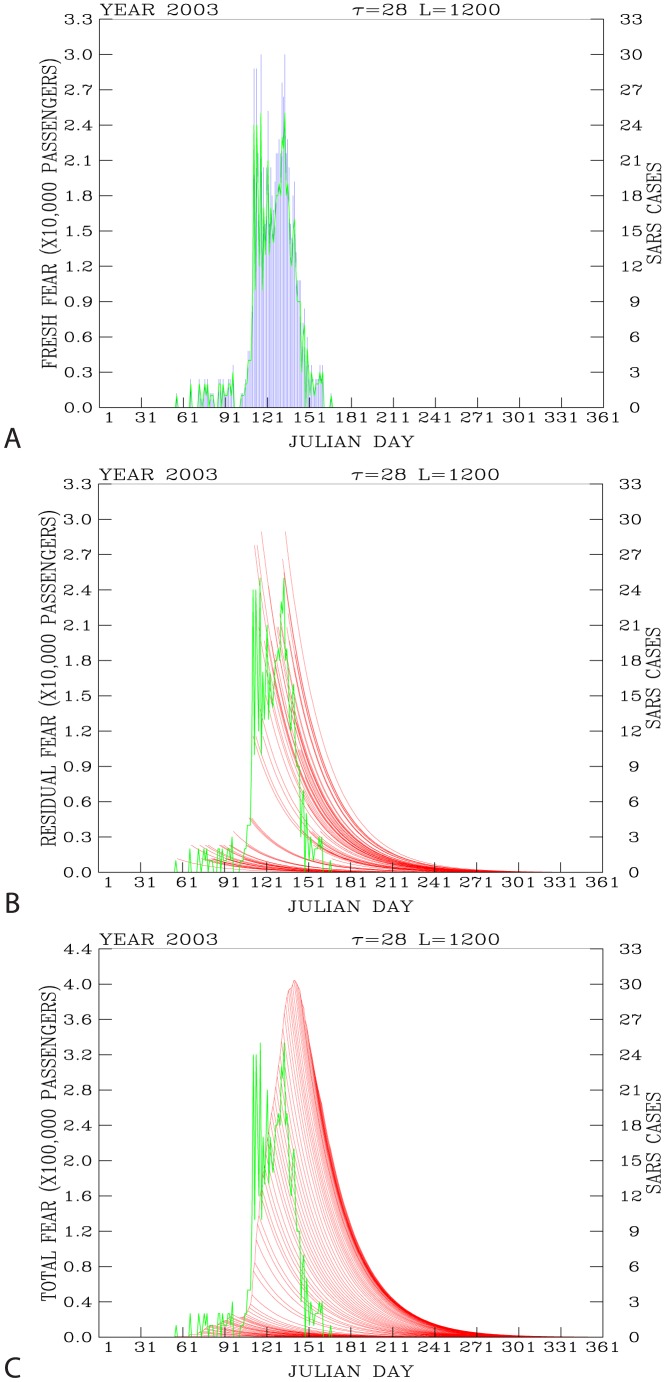
Time-series distribution of the fresh fear (blue lines in A), the residual fear (red curves in B), and the total fear (red curves in C). The daily reported new SARS cases are shown as green curves.

The first daily reported SARS case appeared on 25 February 2003 (Julian day 56; Figure 4A). It is a single case. The second daily reported 2 new SARS cases were made 10 days later, on 7 March (Julian day 66). Then, 6 days later, the third daily reported 2 new SARS cases were announced on 13 March (Julian day 72). After that day, new SARS cases were reported almost daily from 14 March to 9 June (Julian day 160). Significant increase of the daily reported new SARS cases from 4 per day to 24 per day had occurred in a 5-day period, from 17 (Julian day 107) to 21 April (Julian day 111). After 22 April (Julian day 112), the daily reported new SARS cases had maintained between 10 per day and 25 per day until 13 May (Julian day 133).

For reasons still unknown, the daily reported SARS cases started to decrease after 14 May (Julian day 134). On 15 June (Julian day 166), the last daily reported single SARS case were made, and no more SARS cases were announced after that day. Hence, for about 111 days, people's life were shadowed by the daily announcement of new reported SARS cases. It was a fear that was very difficult to get away, and you did not know when this fear would end. As a result, people's fear for contacting the SARS virus linger much longer, until about Julian day 300 (27 October) when the daily underground ridership returns to its normal daily ridership before the SARS epidemic.

### 3. The Fresh Fear, Residual Fear, and Total Fear

Equation 5 and Eqn. 10 show equations for modeling the fresh fear with respect to the daily reported SARS cases. Figure 4A shows calculated results during the SARS period with the loss of underground ridership *L* = 1200 for each reported SARS case.

Since The fresh fear 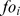 is directly proportional to the total daily reported SARS cases 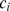 (Eqn. 10), variations of 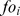 are in phase with 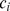.

As such, the maximum reduction due to the daily fresh fear incurred by the newly reported SARS cases are close to 30,000 underground ridership per day. Most of the fresh fears are developed between Julian days 91 (1 April) and 160 (9 June).

The dissipation of each fresh fear to the subsequent days, the residual fear, is modeled according to Eqn. 4. Figure 4B shows calculated time-series distribution of the residual fear with the lifetime of the fresh fear 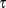 = 28 days. Here we see an ensemble of dissipation of daily fresh fear from the SARS peak period (Julian days 91–160) to the following days.

Hence, the resulted daily total fear according to Eqn. 6 and Eqn. 7 is shown in Figure 4C. The peak of the total fear is the loss of about 400,000 underground ridership per day, which occurs about 10 days after the peak of the daily reported SARS cases.

### 4. The Underground Ridership During the SARS Year

**Figure 5:**
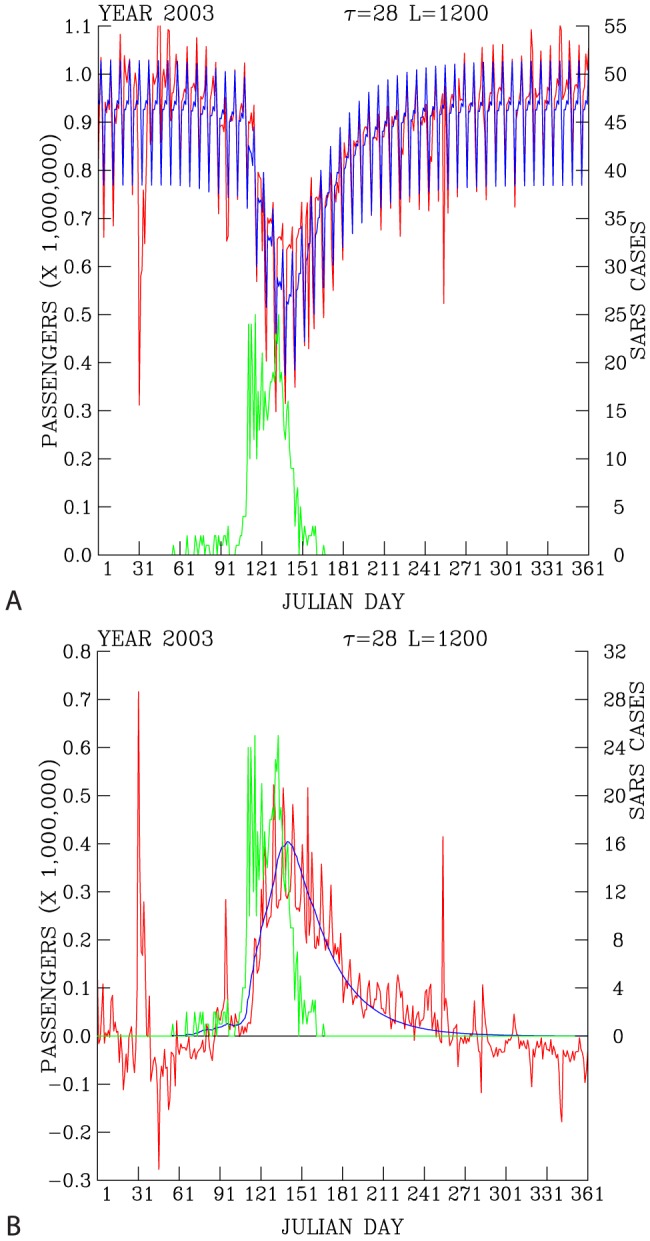
Predicted (blue curve) and actual (red curve) daily ridership (A). Difference in the change of actual daily ridership with respect to the statistical prediction (statistical model-2003 daily ridership; red curve) and the dynamical model prediction (dynamic model - 2003 daily ridership; blue curve) (**B**). Daily reported SARS cases are shown as green curves. Both data are for 2003.

Hence, in a sharp contrast with the normal underground usage during 2001, 2002, 2004, and 2005, the daily ridership in 2003 shows anomalously high loss of ridership from about day 60 to days 120–150, when the maximum reduction of daily ridership of half a million were occurred (Figure 5). About 50% of daily ridership was lost during peak of the 2003 SARS periods. This period concurs with the SARS outbreaks in Taiwan (10, 11).

Figure 5A compares the time evolution of SARS cases and the wane and the wax of the daily underground ridership. The peak of the reduction in the daily ridership occurred after the peak of the reported probable SARS cases.

While the reported SARS cases drop sharply during days 151–181, the returns of the ridership to the underground appear to be at a slow pace during days 151–271.

Predicted loss of the daily underground ridership and its comparison with the actual ridership are shown in Figure 5B. The sharp response in daily ridership following the increase of the reported SARS cases, and the slow return of the ridership after the peak of the SARS cases is well reproduced by the model, Figure 5B.

The close agreement between the model and actual underground ridership indicate that the model can successfully reproduce the daily underground ridership during the 2003 SARS epidemics in Taiwan.

### 5. Sensitivity of Underground Ridership to Reported SARS Cases

Two parameters are keys to the predicted underground ridership with respect to the daily reported SARS cases: Instantaneous ridership loss rate (*L*) per reported SARS case, and the *e*-folding time (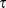) indicating the dissipation of the fresh fear to subsequent days.

**Figure 6:**
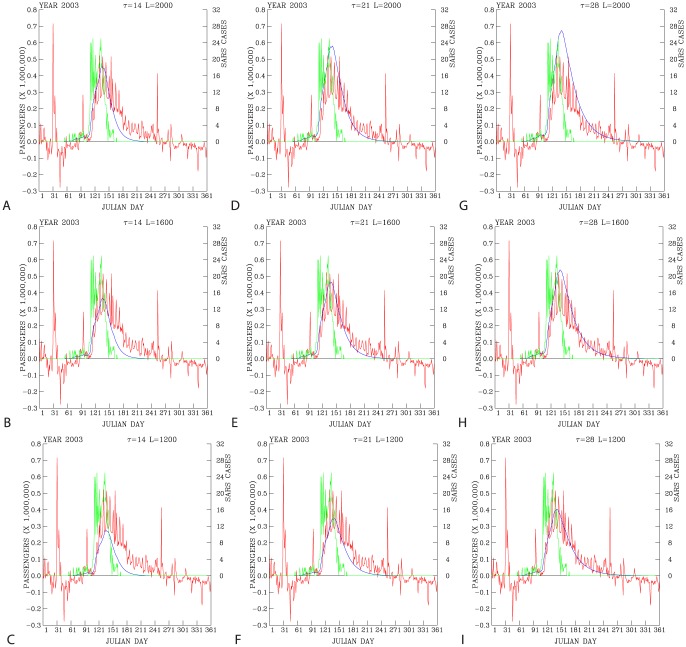
Difference in the actual daily ridership (red curves) and the predicted daily ridership (blue curves) with respect to a combination of three instantaneous passenger reduction rates (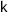) and three passenger 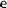-folding time (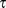) scales. For plots in the columns from the left to the right showing 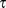 = 14, 21, and 28 days, respectively. For plots in the rows from the bottom to the top showing 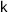 = 1200, 1600, and 2000 ridership loss rates per reported SARS case, respectively. Green curves show daily reported SARS cases. These data are shown for 2003.

Figure 6 shows tests of various values of these two parameters. For the same *e*-folding time (the periods that perceived risk lasts), for example 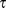 = 14 days (Figures 6A–C), the larger the daily ridership loss rates *L* per reported SARS case (degree of shocks to the public), the deeper the reduction in the underground ridership will be resulted. But the time to return to the normal daily ridership is similar for different ridership loss rates after passing the peaks in the SARS cases. These results indicate that, if the time scales of public perception to each reported SARS case are the same, then the impact on the loss of underground passengers will be limited to the days close to the peak of the reported SARS cases.

On the other hand, if the passenger loss rates are the same, for example *k* = 1200 (Figures 6A, 6D, and 6G), then the longer the *e*-folding time scale 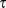, the slower the return of the underground ridership to the normal. A long *e*-folding time scale also results in a large accumulated loss due to the accumulated effects from previous days (Figure 6G). Hence, long period of the public perception of the risk associated with the reported SARS cases is likely to cause the long-lasting impact on the behavior of people and their willingness to use the underground.

We have also computed root mean square errors between the actual daily ridership and the predicted daily ridership for a range of 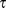 and *L* shown in Figure 6. The smallest value of these root mean square errors are found when 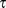 = 28 and *L* = 1200.

The underground daily ridership shows no distinctive drop from mid November 2002 (Figure 2B, started from Julian Day 305) to late February 2003 (Figure 5A, Julian Day 59). This indicates that the underground usage hasn't been directly impacted by reports of the SARS cases occurred abroad until the cases were reported domestically. We speculate that the news report about the outbreak in other places where the diseases traveled from Asia as one reason why ridership in Taipei did not resume back to normal levels even after the epidemic had ended in Taipei on 15 June 2003. Also, the slow return of people from quarantine, sickness, and been away from the work to continuously care for those struck by this epidemic. These are additional factors that may explain why riderships in Taipei did not resume back to normal levels even after the epidemic has ended.

We note that a total of about 131,132 people were quarantined during March–July 2003 [12]. Hence, the effect of people being sick and dying (including the caregivers being away from work and caring for the sick) can have influence on daily ridership. In other words, the fear factor may also implicitly represent effect from people being sick and by people being home caring for the sick. The Level A quarantine was started on 18 March, for those who had been in close contact with a SARS patient were quarantined from 10–14 days. These people include health-care workers, (1751), family members (6663), co-workers and friends (4351), classmates and teachers (14,919), passengers sitting adjacent to SARS patients (1380), discharged suspect and probable SARS patient (1796), and others (19i,459). A total of 50,319 people were Level A quarantined. The Level B quarantine, stared on 28 April, applied to those traveled from SARS-related areas. A total of 80,813 people were Level B quarantined for 10 days.

## Summary

In this work we show that the dynamics of the Taipei underground usage during the 2003 SARS epidemic in Taiwan are closely linked to the daily wax and wane of the reported probable SARS cases.

Our model shows that each reported SARS case results in an immediate loss of about 1200 underground ridership (the fresh fear), reflecting the public fear of immediate risk associated with the intense report of the SARS outbreaks and their reluctance in using the underground system.

The public perception of the risk propagates and exponentially decays to the following days with an *e*-folding time of about 28 days (the residual fear). This duration of time reflects the perception of the risk perceived by the normal underground passengers. Our study shows that the longer the *e*-folding time (perception of the risk), the slower the return of the underground ridership.

A huge loss of the underground ridership but with a short *e*-folding time results in predicted passengers returning to the underground system sooner than what had occurred. The combination of the immediate passenger loss rates (the fresh fear) and their impacts propagates to the following days (the residual fear) resulting in the occurrence of the peak of the ridership loss later than the peak of the reported SARS cases. About 50% of daily ridership was lost during the peak of the 2003 SARS periods, compared with the loss of 80% daily ridership during the closure of the underground system after Typhoon Nari, the loss of 50–70% ridership due to the closure of the governmental offices and schools during typhoon periods, and the loss of 60% daily ridership during Chinese New Year holidays.

Since social distancing measures have been shown to be important for containing an emerging disease [4], [19], [16], [20], [21] our results is useful in incorporating into the disease spreading models where underground usage is an important connection node for social behaviors. There are other major cities such as Hong Kong, Singapore, and Beijing which all contain massive underground systems and were impacted by the 2003 epidemics [22], [23], [24]. Our model developed here is also useful to compare if similar ridership behaviors found in Taipei are also applicable to these major cities in Asia. In the context of avian flu, the underground ridership occurred under the SARS epidemics may provides us a glimpse on what the general public will response in the wake of next epidemics.

Though the fear of the SARS still linger on in 2004, no reported SARS cases in that year resulted in the normal use of the underground system as seen from the model and the actual daily ridership, Figure 2C. Though no significant changes in ridership have occurred after 2003, the 2003 SARS epidemic does indeed make a turning point in people's behavior in Taiwan. People start wearing masks when traveling with trains, underground system, airplanes, etc, after the 2003 SARS year. People also start carrying masks in their handbag as a precaution in case they need it.
